# *Suaeda salsa SsDHN* Gene Enhances Drought Tolerance in Tobacco (*Nicotiana tabacum*)

**DOI:** 10.3390/plants15030443

**Published:** 2026-01-31

**Authors:** Hui Ma, Zhixin Song, Jiahui Wu, Yuou Song, Jingyi Zhang, Ming Zhong, Jingwei Lin, Shuisen Chen, Hui Li

**Affiliations:** 1 Key Laboratory of Agricultural Biotechnology of Liaoning Province, College of Biosciences and Biotechnology, Shenyang Agricultural University, Shenyang 110866, China; 2 Liaoning Panjin Wetland Ecosystem National Observation and Research Station, Panjin 124221, China

**Keywords:** *SsDHN*, drought stress, ROS-scavenging capability, photosynthesis

## Abstract

Drought stress critically constrains plant development and morphogenesis, representing a substantial challenge to crop production systems. Dehydrins (DHNs), belonging to the late embryogenesis abundant (LEA) protein superfamily, play crucial roles in plant adaptation to environmental stress conditions. Nevertheless, the capacity of *Suaeda salsa* SsDHN protein to confer drought resistance has not been adequately investigated. In the present study, transgenic tobacco lines with constitutive *SsDHN* expression (*SsDHN*-OE) were employed to examine its influence on seedling development under water-limited conditions. Results indicated that constitutive *SsDHN* expression enhanced biomass accumulation, foliar expansion, root elongation, and root surface dimensions in water-stressed seedlings. Moreover, transformed lines demonstrated elevated proline (Pro) accumulation and abscisic acid (ABA) content, augmented antioxidant enzyme activity, and intensified stomatal regulation under stress conditions. Conversely, photoinhibition intensity, chloroplast structural degradation, malondialdehyde (MDA) accumulation, electrolyte leakage, hydrogen peroxide (H_2_O_2_), and superoxide radical (O_2_^−^) concentrations were diminished. Furthermore, transcript abundance of stress-responsive genes—encompassing *NtNCED3*, *NtSnRK2.2*, *NtRD26*, *NtLEA5*, *NtPOD*, *NtSOD*, *NtCAT*, and *NtAPX1*—was markedly increased in *SsDHN*-OE lines experiencing drought stress. Taken together, these findings establish that SsDHN functions as a positive regulator of drought resilience in plants.

## 1. Introduction

Environmental stress factors can impede plant development, morphogenesis and productivity, potentially causing mortality under extreme circumstances [1]. Within the spectrum of environmental stressors, water scarcity or dehydration stress constitutes one of the most critical challenges to global agricultural sustainability [2]. Drought stress independently has resulted in agricultural production losses of approximately $30 billion worldwide throughout the preceding ten years [3]. Drought impairs multiple facets of plant physiology by disrupting essential cellular processes [4]. One of the most significant negative impacts of drought stress on plants is the induction of massive production of reactive oxygen species (ROS), which disrupts cellular redox homeostasis [5]. Chloroplasts are one of the primary sources of ROS under drought stress, where excess excitation energy leads to over-reduction of the electron transport chain and generates reactive oxygen species [6]. Concurrently, drought severely inhibits photosynthesis. Initially, this occurs mainly through the induction of stomatal closure, which limits CO_2_ supply. As the stress intensifies, non-stomatal limitations become the dominant factor [7]. These limitations include photoinhibition and reduced efficiency of Photosystem II (PSII) [8], as well as impairments in overall electron transport and carbon metabolism [9]. The burst of ROS and the damage to the photosynthetic apparatus often form a vicious cycle: photosynthetic damage leads to increased ROS production, while ROS further exacerbates oxidative damage to the photosynthetic machinery [10]. Consequently, developing robust approaches to strengthen crop drought resilience for achieving optimal water utilization and maximum productivity represents a pressing necessity. Hence, characterization of genes linked to environmental stress tolerance constitutes a critical priority in elucidating molecular pathways underlying plant stress adaptation.

For persistence under adverse environmental conditions, plants have developed specialized adaptive systems to minimize cellular water depletion, referred to as dehydration avoidance. Late embryogenesis abundant (LEA) proteins represent a key mechanism mediating water stress resistance. LEA proteins comprise an extensive superfamily of water-soluble proteins, initially discovered during terminal seed development phases and subsequently demonstrated to exhibit widespread distribution throughout numerous plant taxa and tissue types. These proteins execute essential functions in plant ontogeny and adaptation to environmental stress [11,12]. LEA proteins are categorized into different subfamilies according to their conserved motifs and structural properties [13]. Among them, dehydrins (DHNs) are members of group II of the LEA proteins and fulfill essential functions in augmenting plant stress resilience [14]. For example, overexpression of *ZmDHN3* improved the resistance to drought stress in maize [15]. Constitutive expression of *Prunus mume* dehydrin genes (*PmLEA10*, *PmLEA19*, *PmLEA20* and *PmLEA29*) enhanced chilling resistance in tobacco [16], and heterologous introduction of *CsLEA1* elevated freezing tolerance in *Escherichia coli* and yeast [17]. Additional cases encompass the cold-inducible wheat dehydrin *wzy1-2* [18] and *SiLEA4*, which provides low-temperature protection in microorganisms and plants [19]. In maize, *DHNs* are critical for defense mechanisms against diverse abiotic stressors: *ZmDHN13* augments copper resistance in yeast and tobacco through metal chelation and ROS reduction [20]; *ZmDHN2b* constitutive expression elevates chilling stress resilience [21]; and *ZmDHN11* strengthens osmotic stress resistance [22]. Correspondingly, wheat *DHN-5* amplifies salinity and osmotic stress tolerance in *Arabidopsis* [23,24], whereas banana *HbDHN1* and *HbDHN2* strengthen drought and osmotic stress resilience [25]. Furthermore, sorghum *SbDHN1* confers protection under elevated-temperature and osmotic stress conditions [26], and pepper *CaDHN3* enhances salinity and drought tolerance through ROS signaling pathways [27]. Overexpression of *PtrDHN-3* enhances salt tolerance in *Arabidopsis* by elevating antioxidant enzyme activity [28], and soybean *GmDHN9* increases drought tolerance [29].

*Suaeda salsa* (L.) Pall. is an annual herb of the Chenopodiaceae family that mainly grows in coastal wetlands, deserts, and other saline environments [30,31]. The salt-tolerant species *Suaeda salsa*, commonly used as a bioindicator of saline soils, serves as a model for studying plant adaptation to salt and drought. DHNs are known to contribute significantly to plant stress resistance. However, the specific function of the *S. salsa* dehydrin, *SsDHN*, under drought conditions remains unclear. Building on our previous work demonstrating its role in salt tolerance in tobacco [32,33], we hypothesized that *SsDHN* might also enhance drought resistance. To test this, we generated and analyzed transgenic tobacco plants overexpressing the *SsDHN* gene, assessing their response to drought stress. This study elucidates the function of *SsDHN* under drought stress and provides a foundation for further research into DHN-mediated abiotic stress tolerance.

## 2. Results

### 2.1. Overexpression of SsDHN Enhanced Drought Tolerance in Tobacco

**Figure 1:**
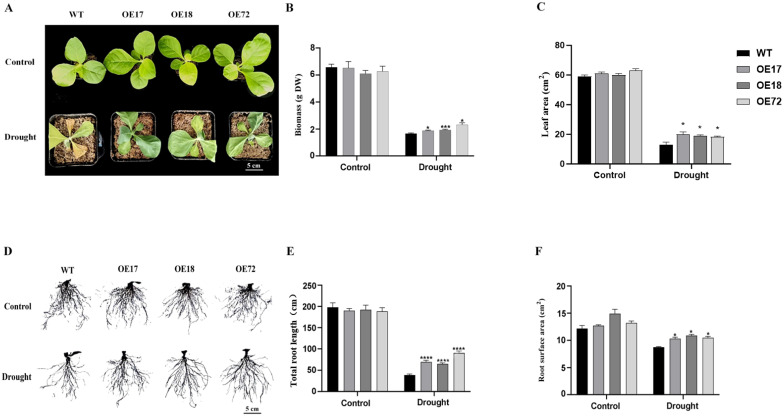
*SsDHN* overexpression enhanced drought tolerance in tobacco plants, as evidenced by (**A**) phenotypic differences between WT and transgenic lines after 15 d of drought. Associated growth parameters were quantified, including (**B**) biomass, (**C**) leaf area, and-based on the root phenotype shown in (**D**,**E**) root length and (**F**) root surface area. Data represent mean ± SD [n = 3 biological replicates (with at least 10 plants per replicate)]. * *p* < 0.05, *** *p* < 0.001, **** *p* < 0.0001 (Student’s *t*-test).

**Figure 2:**
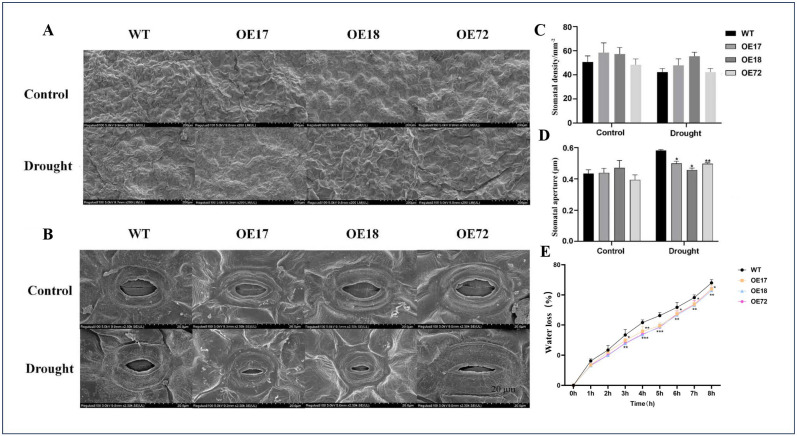
Constitutive *SsDHN* expression altered stomatal properties in tobacco leaves under drought stress. Analysis of stomatal density (**A**) and stomatal aperture (**B**) via scanning electron microscopy in WT and *SsDHN* constitutive expression seedlings. Scale bar = 20 μm. Measurements of stomatal density (**C**), stomatal aperture (**D**) and leaf water loss (**E**) in WT and *SsDHN* constitutive expression seedlings. Data presented as mean ± SD [n = 3 biological replicates (with at least 10 plants per replicate)]. * *p* < 0.05, ** *p* < 0.01, *** *p* < 0.001 (Student’s *t*-test).

Under well-watered conditions, no significant differences in biomass, leaf area, root length, or root surface area were observed between wild-type (WT) and *SsDHN*-overexpressing plants. Drought stress markedly inhibited overall seedling growth and leaf development (Figure 1A), yet the transgenic lines exhibited significantly greater values for all measured growth parameters compared to the WT (Figure 1B–F). Stomatal density remained unchanged across genotypes under both control and drought conditions (Figure 2A,B). However, under drought stress, the stomatal apertures were considerably narrower in *SsDHN*-overexpressing plants than in the WT (Figure 2C,D). Correspondingly, transgenic leaves displayed a slower rate of water loss (Figure 2E). Furthermore, the *SsDHN* overexpression plants accumulated substantially more abscisic acid (ABA) than the WT plants under drought stress (Figure S1). These results indicate that *SsDHN* overexpression enhances drought tolerance, at least in part, by reducing water transpiration through stomatal regulation.

### 2.2. Overexpression of SsDHN Reduces Electrolytic Leakage and Lipid Peroxidation Under Drought Stress

**Figure 3:**
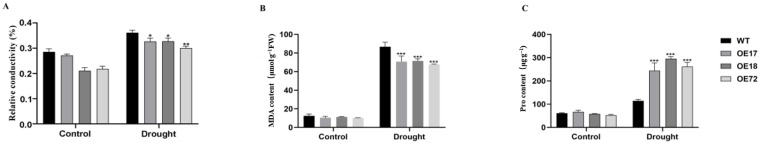
*SsDHN* gene reduces MDA content and relative conductivity and increases proline content after drought treatment. (**A**) The relative conductivity was analyzed after drought treatment. (**B**) The content of MDA was analyzed after drought treatment. (**C**) The content of proline was analyzed after drought treatment. Data represent mean ± SD [n = 3 biological replicates (with at least 10 plants per replicate)]. * *p* < 0.05, ** *p* < 0.01, *** *p* < 0.001 (Student’s *t*-test).

Under irrigated conditions, no significant differences were observed between WT and *SsDHN*-overexpressing plants in relative conductivity, malondialdehyde (MDA) content, or proline (Pro) levels. Following drought stress, relative conductivity and MDA content increased in both genotypes. However, compared to the WT, the three transgenic lines (*SsDHN*-OE17, -OE18, -OE72) showed significantly lower relative conductivity, whereas Pro accumulation was notably elevated in *SsDHN* constitutive expression plants relative to WT (Figure 3). Consequently, constitutive expression of the *SsDHN* gene under drought conditions markedly reduced MDA accumulation and electrolyte conductivity while augmenting Pro concentration.

### 2.3. Overexpression of SsDHN Reduced Reactive Oxygen Species (ROS) Accumulation in Leaves Under Drought Stress

**Figure 4:**
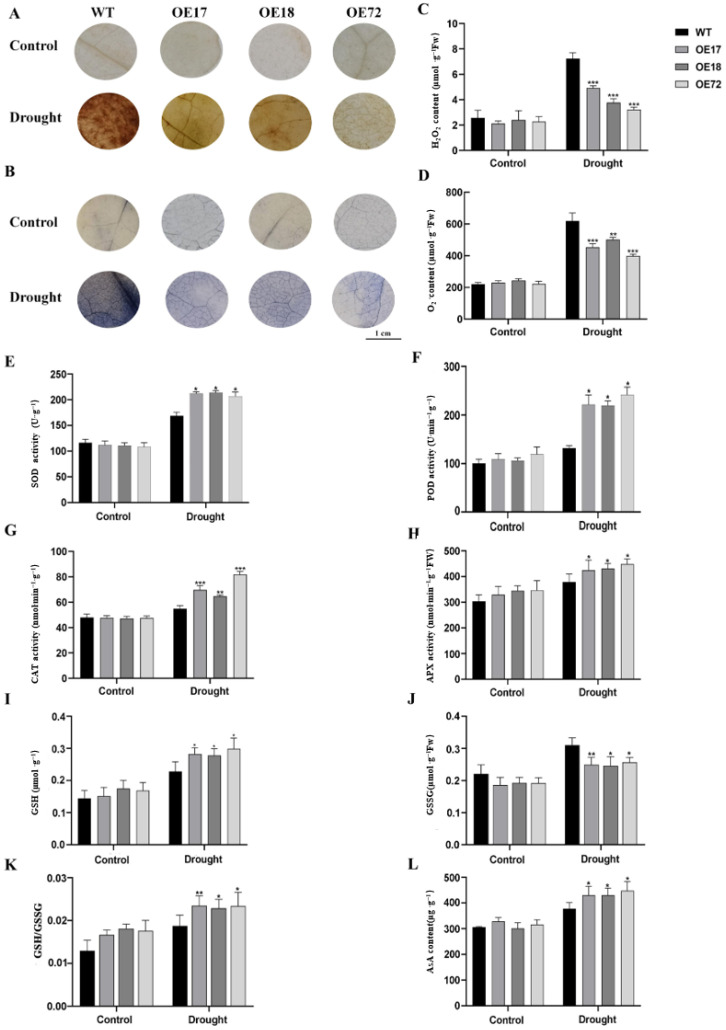
Constitutive *SsDHN* expression reduced ROS buildup and enhanced the ROS-detoxification capacity in transgenic tobacco under drought stress. H_2_O_2_ (**A**) and O_2_^−^ (**B**) were visualized in situ through DAB and NBT histochemical staining, respectively. Associated quantitative determinations include H_2_O_2_ levels (**C**), O_2_^−^ levels (**D**), and the catalytic activities of principal antioxidant enzymes: SOD (**E**), POD (**F**), CAT (**G**), and APX (**H**). Furthermore, redox metabolite concentrations were evaluated: GSH (**I**), GSSG (**J**), the GSH/GSSG ratio (**K**), and AsA concentration (**L**). Values represent mean ± SD [n = 3 biological replicates (with at least 10 plants per replicate)]. * *p* < 0.05, ** *p* < 0.01, *** *p* < 0.001 (Student’s *t*-test).

Under drought stress, histochemical staining with nitro blue tetrazolium (NBT) and 3,3′-diaminobenzidine (DAB) revealed deeper staining in WT leaves compared to *SsDHN*-overexpressing plants, indicating greater accumulation of superoxide radical (O_2_^−^) and hydrogen peroxide (H_2_O_2_), in the WT (Figure 4A,B). Quantitative assays confirmed that the contents of both O_2_^−^ and H_2_O_2_ were significantly lower in transgenic plants under drought, while no differences were observed under control conditions (Figure 4C,D). Similarly, antioxidant enzyme activities of superoxide dismutase (SOD), ascorbate peroxidase (APX), peroxidase (POD), and catalase (CAT) did not differ between genotypes under normal conditions but were markedly higher in *SsDHN*-overexpressing plants during drought stress. Furthermore, transgenic plants exhibited increased levels of reduced glutathione (GSH) and ascorbic acid (AsA), a higher GSH/GSSG ratio, and a lower oxidized glutathione (GSSG) content compared to the WT under drought (Figure 4E–L). These results demonstrate that *SsDHN* overexpression enhances the antioxidant capacity in tobacco by boosting both enzymatic and non-enzymatic systems, thereby improving ROS scavenging and alleviating membrane damage under drought stress.

### 2.4. Constitutive SsDHN Expression Augmented Photosynthetic Pigment Accumulation and Photosynthetic Efficiency Under Drought Stress

**Figure 5:**
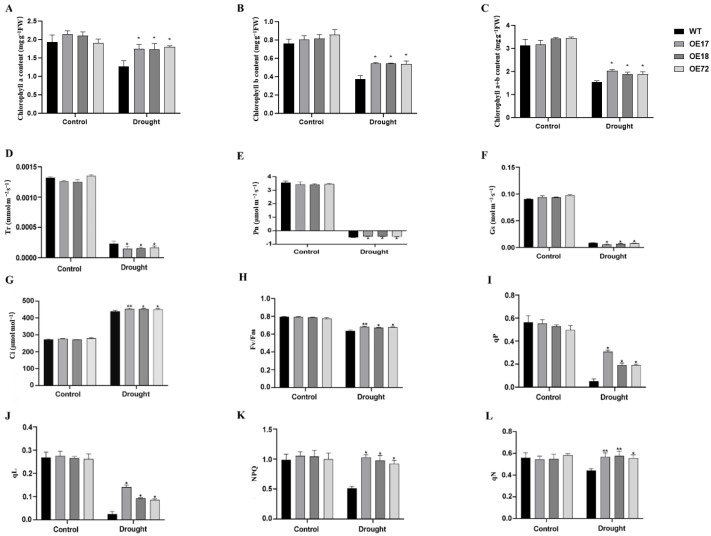
Constitutive *SsDHN* expression regulates photosynthetic pigment levels and key photosynthetic parameters in tobacco foliage experiencing 15-day drought stress. The measured metrics include (**A**) chlorophyll *a*, (**B**) chlorophyll *b*, and (**C**) total chlorophyll levels, (**D**) transpiration rate, (**E**) net photosynthetic rate, (**F**) stomatal conductance, and (**G**) intercellular CO_2_ concentration, (**H**) Fv/Fm, (**I**) qP, (**J**) qL, (**K**) NPQ, and (**L**) qN in both WT and *SsDHN*-OE seedlings. Data presented as mean ± SD [n = 3 biological replicates (with at least 10 plants per replicate)]. * *p* < 0.05, ** *p* < 0.01 (Student’s *t*-test).

During water deficit exposure, chlorophyll *a*, chlorophyll *b*, and aggregate chlorophyll concentrations declined in tobacco seedlings. Nevertheless, transformed lines retained substantially elevated concentrations of these pigments in comparison to WT genotypes (Figure 5A–C). The net CO_2_ assimilation (Pn), transpirational water loss (Tr), and stomatal conductance (Gs) in *SsDHN* constitutive expression plants were substantially reduced compared to those in WT genotypes, whereas internal CO_2_ levels (Ci) in *SsDHN* constitutive expression plants were markedly elevated relative to those in WT genotypes (Figure 5D–G). The maximal photochemical quantum yield of PSII (Fv/Fm) demonstrated equivalence between WT and transformed lines under control regimes. Nevertheless, subsequent to drought imposition, a pronounced elevation in Fv/Fm was detected in transgenic tobacco genotypes compared to the WT (Figure 5H). Correspondingly, the chlorophyll fluorescence indices qN, NPQ, qP, and qL exhibited no substantial differential under standard conditions but were conspicuously amplified in transgenic genotypes compared to the WT under drought stress (Figure 5I–L). These observations indicate that *SsDHN* expression facilitates preservation of elevated photosynthetic electron transport efficiency under water-limited conditions.

### 2.5. Effects of SsDHN on Chloroplast Ultrastructure in Tobacco Leaves Under Drought Stress

**Figure 6:**
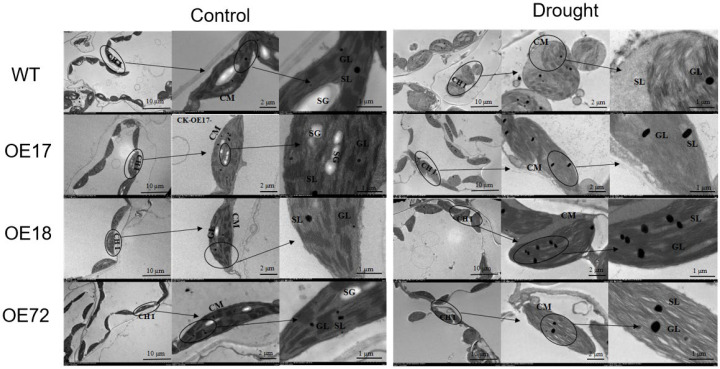
Influence of *SsDHN* on chloroplast ultrastructural organization in tobacco seedlings exposed to 15 days of drought stress. Scale bars correspond to 10, 2, and 1 µm. Abbreviations: CHl, chloroplasts; CM, chloroplast membrane; SG, starch grains; GL, grana lamellae; SL, stroma lamellae.

Under standard conditions, chloroplasts in both WT and transformed lines were positioned adjacent to the plasma membrane, displaying a fusiform morphology with clearly demarcated inner and outer envelope membranes. Thylakoid stacking was systematically arranged, and the stromal lamellae demonstrated compact organization. Plentiful starch deposits, representing vigorous photosynthetic activity, additionally validated normal chloroplast functionality. Subsequent to drought stress imposition, chloroplasts in WT genotypes adopted spherical and distended configurations, forfeiting their membrane-proximal positioning. The majority of chloroplasts displayed degradation of plastid envelope membranes, increased vacuolar formation emerged in the internal disrupted regions of chloroplasts, stromal lamellae underwent fragmentation and disorganization, granal lamellae suffered complete structural collapse and disorder, starch grain biosynthesis ceased, and osmiophilic globule abundance intensified. Following drought stress exposure, the chloroplasts of transgenic tobacco transitioned from spindle-shaped to elongated morphology and remained positioned along the plasma membrane, though the chloroplasts no longer maintained a dense arrangement, and occasional chloroplasts adopted spherical and distended forms. The stromal lamella and grana lamella were not broken, but the boundaries were blurred. The structure of the grana lamella becomes loose, smaller vacuoles appear in the chloroplast, and a small part of the chloroplast membrane dissolves. Drought stress resulted in varying degrees of damage to the chloroplast ultrastructure of tobacco plants. WT tobacco exhibited more severe disruption of chloroplast membranes and internal lamellae, while transgenic lines maintained greater structural integrity in both the lamellar and membrane systems (Figure 6). These findings indicate that *SsDHN* exerts a protective effect on the chloroplast ultrastructure under drought stress.

### 2.6. Overexpression of SsDHN Increased the Expression of Stress-Related Genes Under Drought Stress

**Figure 7:**
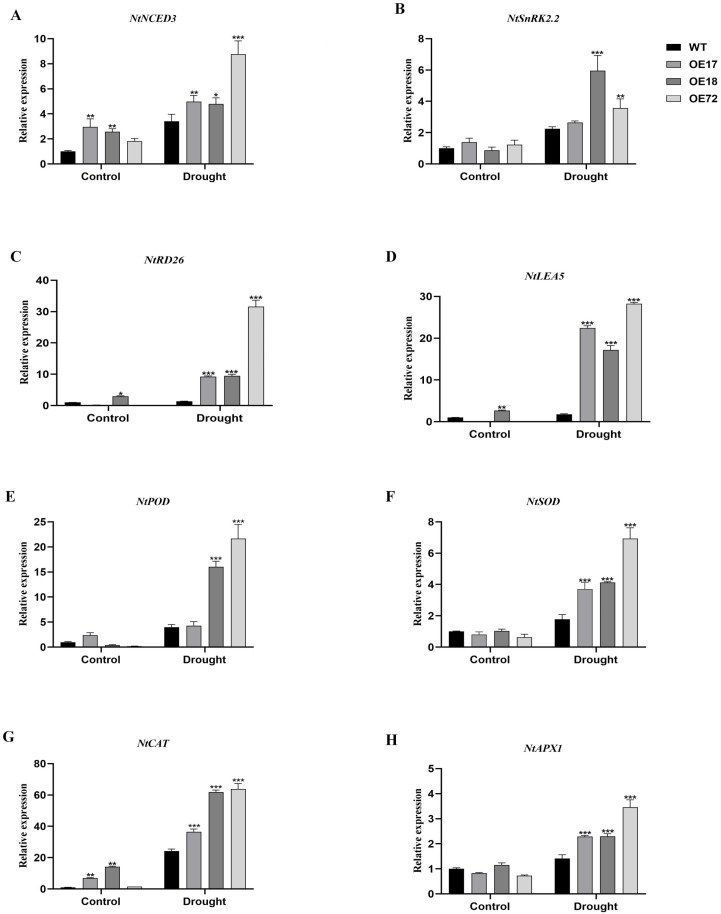
Constitutive expression of the *SsDHN* gene up-regulated the transcript abundance of stress-responsive genes in transgenic tobacco under drought stress, encompassing (**A**) *NtNCED3*, (**B**) *NtSnRK2.2*, (**C**) *NtRD26*, (**D**) *NtLEA5*, (**E**) *NtPOD*, (**F**) *NtSOD*, (**G**) *NtCAT*, and (**H**) *NtAPX1*. Data are expressed as mean ± SD from three independent experiments. * *p* < 0.05, ** *p* < 0.01, *** *p* < 0.001 (Student’s *t*-test).

To elucidate the underlying molecular mechanisms contributing to the enhanced tolerance to drought stress in *SsDHN* constitutive expression plants, we examined the transcript abundances of stress-responsive genes, encompassing *NtNCED3*, *NtSnRK2.2*, *NtRD26*, *NtLEA5*, *NtPOD*, *NtSOD*, *NtCAT* and *NtAPX1*. Under control regimes, there was no substantial differential in the stress-responsive genes *NtNCED3*, *NtSnRK2.2*, *NtRD26*, *NtLEA5*, *NtPOD*, *NtSOD*, *NtCAT* and *NtAPX1* between WT and SsDHN constitutive expression plants. Nevertheless, the transcript levels of *NtNCED3*, *NtSnRK2.2*, *NtRD26*, *NtLEA5*, *NtPOD*, *NtSOD*, *NtCAT* and *NtAPX1* were markedly elevated under drought stress. In comparison to the WT genotypes, the *SsDHN* constitutive expression plants demonstrated elevated transcript abundances of *NtNCED3*, *NtSnRK2.2*, *NtRD26*, *NtLEA5*, *NtPOD*, *NtSOD*, *NtCAT* and *NtAPX1* under drought treatment (Figure 7). These observations indicate that constitutive expression of the *SsDHN* gene could facilitate the transcript induction of stress-responsive genes under drought stress.

## 3. Discussion

LEA proteins constitute a ubiquitous class of proteins in plants and fulfill a critical function in adversity resistance [12]. DHN is a thermostable protein belonging to a subfamily of the LEA protein family, characterized by high hydrophilicity and stress responsiveness [34]. It has been documented that the transcript induction of the *DHN* gene fulfills an essential function in plant adaptation to abiotic stresses, including drought, salinity and low temperature [35,36]. *DHN* genes are promising for use in genetic engineering to enhance plant stress tolerance. Studying the molecular mechanisms of increased stress tolerance in these transgenic plants is of great interest. Nevertheless, the function of the *Suaeda salsa SsDHN* gene in mediating the drought response remains inadequately characterized. To examine the functionality of *SsDHN*, we generated transgenic tobacco with constitutive *SsDHN* expression. Drought stress resulted in elevated biomass, expanded leaf area, extended root length and increased root surface area in *SsDHN* constitutive expression plants in comparison to WT genotypes (Figure 1). Stomata fulfill a pivotal function in plant perception of environmental fluctuations, encompassing drought and salinity stress [37]. Modulating stomatal pore dimensions to minimize water depletion through transpiration constitutes a critical determinant of drought resistance [38]. In this investigation, we found that the *SsDHN* constitutive expression plants had much narrower stomatal apertures than the WT plants under drought stress. Furthermore, the *SsDHN* constitutive expression plants demonstrated a decelerated water loss rate compared to the WT under drought stress (Figure 2), which was concordant with stomatal closure behavior. Drought stress inflicts damage to plants predominantly through osmotic stress and oxidative stress. Consequently, MDA concentration and electrolyte conductivity are frequently employed to reflect membrane integrity. Plants generate osmotic adjustment factors such as Pro to alleviate the osmotic stress [39,40]. Under drought stress, *SsDHN* constitutive expression diminished relative electrolyte efflux and MDA concentration while augmenting Pro concentrations (Figure 3). These observations suggest that *SsDHN* enhances drought tolerance in tobacco, presumably by facilitating proline accumulation to ameliorate membrane damage under stress conditions.

Drought stress leads to the overproduction of ROS, which in turn imposes oxidative damage on intracellular components [41]. ROS are intermediates produced during O_2_ reduction and predominantly include O_2_^−^ and H_2_O_2_ [42]. DAB and NBT staining are validated methods for detecting H_2_O_2_ and O_2_^−^, respectively [43]. Concordant with the observed reductions in MDA concentration and electrolyte leakage (Figure 3), *SsDHN* constitutive expression under drought stress resulted in diminished accumulation of both H_2_O_2_ and O_2_^−^ (Figure 4A–D). Plants deploy enzymatic and non-enzymatic antioxidant systems to eliminate ROS and ameliorate oxidative damage [44]. Analogous functions have been documented for other dehydrins; for instance, *CaDHN3* enhances salt and drought tolerance in *Arabidopsis* by restricting ROS accumulation [27], and *ZmDHN15* improves cold stress tolerance [45]. In this investigation, transgenic plants demonstrated substantially elevated activities of key antioxidant enzymes and augmented concentrations of non-enzymatic antioxidants under drought regimes (Figure 4E–L), which correlated with diminished ROS. These outcomes indicate that *SsDHN* improves drought tolerance in tobacco by augmenting the ROS-scavenging capacity, thereby reducing oxidative stress.

Drought stress can inflict severe photosystem inhibition, culminating in photodamage and degradation of chloroplasts. It has been demonstrated that the knockdown of the *StTST3.1* gene results in diminished chlorophyll concentration and compromised photosynthesis, which affects the development of potato plants [46]. In this investigation, constitutive expression of *SsDHN* in tobacco augmented chlorophyll concentration and photosynthetic capacity under drought stress (Figure 5). Transmission electron microscopy disclosed that drought stress severely compromised chloroplast morphology and ultrastructure; nevertheless, this damage was considerably ameliorated in *SsDHN* transgenic plants (Figure 6). Such structural deterioration may be associated with ROS-induced lipid peroxidation [47]. These observations suggest that *SsDHN* presumably enhances drought tolerance in tobacco by protecting the photosynthetic apparatus. However, whether *SsDHN* directly stabilizes thylakoid membrane proteins or indirectly protects the photosynthetic system by reducing ROS remains to be further studied.

It is postulated that the enhanced abiotic stress tolerance is predominantly attributed to the substantially elevated transcript abundance of abiotic stress response genes (*CaCAT2*, *CaSOD*, *CaAPX1* and *CaPOD*) under salt and drought stress [27]. In this investigation, *SsDHN* constitutive expression under drought stress markedly up-regulated the transcript levels of genes participating in ABA biosynthesis (*NtNCED3*), ABA signaling (*NtSnRK2.2*), stress response (*NtRD26*, *NtLEA5*), and antioxidant defense (*NtPOD*, *NtSOD*, *NtCAT*, *NtAPX1*) (Figure 7). Consistent with its role in stress signaling, overexpression of *SsDHN* led to a significant increase in ABA accumulation under drought stress compared to WT plants (Figure S1), which likely contributed to their improved drought resistance. These outcomes suggest that *SsDHN* enhances drought tolerance, potentially by modulating these key stress-responsive genes. Considering the central functions of *NCED* in ABA synthesis [48,49] and SnRK2 in ABA perception [50], our observations indicate that *SsDHN* may function through an ABA-dependent signaling pathway to positively regulate the plant’s drought response. Additional experimentation is required to comprehensively validate this mechanism.

## 4. Conclusions

**Figure 8:**
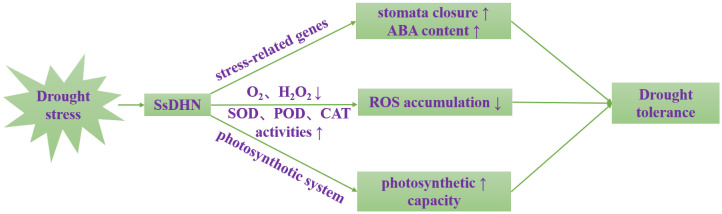
Schematic diagram of the mechanism of *SsDHN* regulating drought tolerance in tobacco.

In summary, we have identified *SsDHN* as a novel dehydrin gene that confers dual advantages: enhanced oxidative stress tolerance and maintained photosynthetic integrity (Figure 8). These findings position *SsDHN* as a prime candidate for molecular breeding, offering a valuable genetic resource for developing crops with comprehensive drought resistance.

## 5. Materials and Methods

### 5.1. Plant Materials and Growth Conditions

The wild-type (WT) genotypes utilized in this investigation were *Nicotiana tabacum* L. cv. NC89. The production of transgenic tobacco lines with constitutive *SsDHN* expression was executed as previously documented [32]. In brief, the CDS region of the *SsDHN* gene was inserted downstream of the CaMV 35S promoter element. For genetic transformation, a 700 bp sequence was inserted into the pBI121 vector, and the resultant construct was introduced into *Agrobacterium tumefaciens* GV3101 for transformation of WT tobacco foliar explants through the leaf disk methodology. Six independent T0 transgenic lines were generated, and three lines (17, 18, and 72), which exhibited the most pronounced *SsDHN* transcript abundance in the T3 generation, were chosen and designated as *SsDHN-OE17*, *SsDHN-OE18*, and *SsDHN-OE72*, respectively. Plants were grown in growth chambers under a 16 h day (at 27 °C), 8 h night (at 23 °C) cycle and 60–70% relative humidity.

### 5.2. Drought Treatment and Measurement of Phenotype-Related Parameters

Tobacco seeds were surface-disinfected and allowed to germinate on MS medium for a 10 d period before being relocated into pots containing a 1:1 mixture of vermiculite and nutrient-enriched soil. Homogeneous 7-week-old T_3_ seedlings from both WT and transgenic lines were selected for drought imposition. The drought-stressed group (≥15 plants) was subjected to complete water withdrawal, while the control group maintained consistent irrigation across the experimental timeframe. Upon completion of drought treatment, substrate particles were washed away from root surfaces, and root systems were harvested post-cleaning. Aboveground tissue dry weight was established after oven-drying specimens at 85 °C for a 72 h period. Leaf surface area was determined using three plants per treatment group (n = 3 biological replicates) through ImageJ version 1.54r software analysis. For root system analysis, roots from 10 individual plants per treatment were spread in a thin layer of water on a blue background and scanned using an EPSON scanner (Seiko Epson Corporation, Suwa, Japan). Images were analyzed for length and diameter using WinRHIZO (Regent Instruments Inc., Quebec, QC, Canada), with the background threshold manually optimized for each image.

### 5.3. Determination of Relative Conductivity, MDA, and Proline Content

MDA levels were established following a published methodology [51] with modifications. Briefly, samples were generated by grinding 0.2 g of fresh leaf tissue in 2 mL of 10% trichloroacetic acid (TCA). The extracted supernatant (2 mL) was mixed with an equal amount of 0.6% thiobarbituric acid (TBA). The mixture underwent heating in boiling water for 15 min, followed by immediate ice cooling. Spectrophotometric readings were taken at 450, 532, and 600 nm, and MDA concentration was calculated according to the standard formula.

Leaf ion leakage was established using a published methodology [52] with slight modifications. Briefly, six leaf disks were placed in 10 mL distilled water at 30 °C for a 2 h period, after which baseline conductivity (EC1) was determined. Samples then underwent boiling for 20 min, were cooled to room temperature, and final conductivity (EC2) was measured. Ion leakage (EL) was calculated using the formula EL = (EC1/EC2) × 100%. Three biological replicates were used per experimental run.

Proline levels were determined using a published methodology [53] with slight alterations. Leaf material (0.5 g) underwent extraction in 5 mL of 3% sulfosalicylic acid at 95 °C for 15 min. The resulting homogenate was centrifuged at 4000× *g* and 4 °C for 10 min. Following centrifugation, the supernatant (2 mL) was mixed with 2 mL of glacial acetic acid plus 2 mL of acid-ninhydrin reagent. This mixture was then subjected to heating in boiling water for 30 min. The mixture underwent extraction with 5 mL of toluene and vigorous mixing, and the toluene layer’s absorbance was determined at 532 nm using a toluene reference.

### 5.4. Assessment of Oxidative Stress and Antioxidant Enzyme Activity

Leaf tissues (0.5 g) from T_3_ transgenic and WT tobacco plants at 7 weeks of age were sampled after a 15 d water deprivation period. Histochemical detection was performed using DAB and NBT post-drought stress [54]. The O_2_^−^ and H_2_O_2_ levels in leaf tissues were determined through Solarbio detection kits following manufacturer’s instructions (Solarbio, Beijing, China). Enzymatic activities of SOD, APX, POD, and CAT were determined through specialized assay kits (Solarbio, Beijing, China), following provided protocols. Similarly, the levels of GSH, GSSG, and AsA were established using appropriate kits (Grace, Suzhou, China). For individual transgenic lines, samples were obtained from no fewer than nine seedlings, with all determinations conducted in triplicate.

### 5.5. Scanning Electron Microscopy and Measurements of Stomatal Aperture

Fresh tobacco leaf material was initially fixed in electron microscopy fixative at room temperature for a 2 h duration, then stored at 4 °C. Post-fixation, samples underwent four washes with 0.1 M phosphate-buffered saline (PBS, pH 6.8; 10 min per wash) before dehydration using a graded ethanol sequence (20 min at each concentration). After additional washing with pure ethanol three times (30 min per wash), samples were transferred into tert-butanol (30 min per transfer). Samples were affixed to metal stubs via carbon tape and then gold-coated using sputter coating. Imaging and visualization were performed on a Hitachi S-3400N scanning electron microscope (Hitachi, Tokyo, Japan). Stomatal opening dimensions were analyzed using ImageJ software. For individual transgenic lines and wild-type controls, no fewer than 10 leaves were examined, with no fewer than 10 viewing fields and 50 stomatal pores analyzed per genotype.

### 5.6. Determination of Photosynthetic Pigment Content and Photosynthetic Parameters

Chlorophyll levels in fresh tobacco leaves were established using a previously published methodology [55]. Leaf samples underwent immersion in 80% acetone and were kept in complete darkness for 48 h. Spectrophotometric measurements were then performed at 663, 645, and 440 nm with 80% acetone serving as the reference blank. Gas exchange parameters—including Tr, Pn, Gs, and Ci—were measured using a Yaxin-1105 portable photosynthesis-fluorescence apparatus (Yaxin, Beijing, China). Measurements occurred on sunny days during the 9:00 to 11:00 a.m. window. For individual lines, three randomly chosen plants were employed, with leaves from similar positions chosen for measurement. Data recording occurred after value stabilization, with three replicate measurements per leaf; mean values represented individual biological replicates. Fluorescence parameters were measured via a Dual-PAM-100 fluorometer (Heinz Walz GmbH, Effeltrich, Germany). For individual treatments, the second-to-last fully expanded leaf was chosen from three randomly selected plants. After a 30 min dark adaptation period, leaves were positioned in the instrument’s measurement chamber for chlorophyll fluorescence assessment. Three measurements were taken per leaf and averaged as one biological replicate.

### 5.7. Observation of Chloroplast Structure Under Transmission Electron Microscope

For preliminary preservation, leaf segments were submerged in a preservative mixture consisting of 1% acetic acid combined with 2.5% glutaraldehyde. Following initial preservation, specimens were progressively dehydrated through an incremental ethanol gradient, encased in epoxy resin matrix, and sliced into ultrathin cross-sections using an ultramicrotome (Leica EM UC7, Vienna, Austria). Upon completion of staining protocols, chloroplast microstructural characteristics were visualized and documented through a Zeiss LSM510 transmission electron microscope (Carl Zeiss AG, Jena, Germany).

### 5.8. Quantitative RT-PCR Analysis

Total cellular RNA was isolated from specimens employing a Plant Total RNA Extraction Kit (TianGen, Beijing, China). In accordance with supplier recommendations, residual chromosomal DNA was eliminated through gDNA Eraser (Takara, Beijing, China) treatment. Complementary DNA synthesis was achieved using the PrimeScript™ RT Kit (TransGen, Beijing, China). Quantitative reverse transcription PCR was executed employing SYBR Premix Ex Taq™ (Takara, Beijing, China) on a Roche LightCycler^®^ 480 II instrument (Roche Diagnostics, Mannheim, Germany), conforming to supplier protocols. *NtActin* was employed as the housekeeping reference gene. To enable gene expression comparisons, relative mRNA abundance of target sequences was computed through the 2^−∆∆CT^ approach [56]. All amplification reactions were run with 3 independent biological replicates. Oligonucleotide primer sequences employed for qRT-PCR are cataloged in Supplementary Table S1.

### 5.9. Determination of ABA Content

Endogenous ABA levels in tobacco leaves were quantified using an enzyme-linked immunosorbent assay (ELISA) kit (Mlbio, Beijing, China). Briefly, leaf tissue (0.1 g fresh weight) was homogenized in 0.2 M phosphate buffer (pH 7.4). The homogenate was centrifuged, and the resulting supernatant was collected for analysis according to the manufacturer’s instructions. Three independent biological replicates were performed.

### 5.10. Statistical Analysis

All measurements are displayed as mean ± standard deviation (SD), calculated from a minimum of 3 independent biological replicates, with each replicate comprising three technical measurements. Statistical differences were evaluated using Student’s *t*-test (* *p* < 0.05, ** *p* < 0.01, *** *p* < 0.001, **** *p* < 0.0001).

## Data Availability

All experimental data supporting this study’s conclusions are available in the manuscript and Supplementary Materials or can be obtained from the corresponding author upon reasonable request.

## Supplementary Materials

The following supporting information can be downloaded at: https://www.mdpi.com/article/10.3390/plants15030443/s1, Figure S1. ABA levels in *SsDHN* overexpression plants. Data represent the mean ± SD (n = 3, * *p* < 0.05 by Student’s *t*-test). Table S1. Primers used for qRT-PCR assay. Table S2. A list of abbreviations and their meanings.
