# Supplementary material for: Suaeda salsa SsDHN Gene Enhances Drought Tolerance in Tobacco (Nicotiana tabacum)
Source: Plants (Basel). 2026 Jan 31;15(3):443. doi: 10.3390/plants15030443 (PMC12899145; doi:10.3390/plants15030443)
Supplement: Supplementary file 1 [file plants-15-00443-s001.zip › plants-4068694-supplementary.pdf]

**Table S1.** Primers used for qRT-PCR assay.

| Primer name | Sequence (5'–3') |
|---|---|
| NtSnRK2.2-F | GGAGTGGTGAGCTTTTTGCTGTTAA |
| NtSnRK2.2-R | GCATTGAGTGACAGTAACTGACTC |
| NtNCED3-F | CACCAGCCCAAGTTATCAATGCCATC |
| NtNCED3-R | TGGTTTTGAAGTGGGAATAGTAG |
| NtSOD-F | CTCCTACCGTCGCCAAAT |
| NtSOD-R | GCCCAACCAAGAGAACCC |
| NtCAT-F | AGGTACCGCTCATTCACACC |
| NtCAT-R | AAGCAAGCTTTTGACCCAGA |
| NtPOD-F | CCTCAGCTTCAAGCATTATGTCCA |
| NtPOD-R | ACCTTTGTAGAAGCATCGGTCCAC |
| NtLEA5-F | TTGAATCTGGGGTTTTGGTT |
| NtLEA5-R | GGAAGCATTGACGAGCTAGG |
| NtAPX1 F | GACATTGCTATCAGACTC |
| NtAPX1-R | CTCCAGTAACTTCAACAG |
| NtRD26-F | GCTCCCAAATGGGACGACCT |
| NtRD26-R | GCATTTGGGGCTCAAAAGGGT |
| Actin2-F | ACCTCTATGGCAACATTGTGCT |
| Actin2-R | CTGGGAGCCAAAGCGGTGATT |

**Table S2.** A list of abbreviations and their meanings.

| Full name | Abbreviation |
|---|---|
| malondialdehyde | MDA |
| proline | Pro |
| reactive oxygen species | ROS |
| nitro blue tetrazolium | NBT |
| 3,3'-diaminobenzidine | DAB |
| superoxide radical | $O_2^-$ |
| and hydrogen peroxide | $H_2O_2$ |
| superoxide dismutase | SOD |
| peroxidase | POD |
| catalase | CAT |
| ascorbate peroxidase | APX |
| glutathione | GSH |
| oxidized glutathione | GSSG |
| ascorbic acid | AsA |
| net $CO_2$ assimilation | Pn |
| transpirational water loss | Tr |
| stomatal conductance | Gs |
| internal $CO_2$ levels | Ci |
| abscisic acid | ABA |

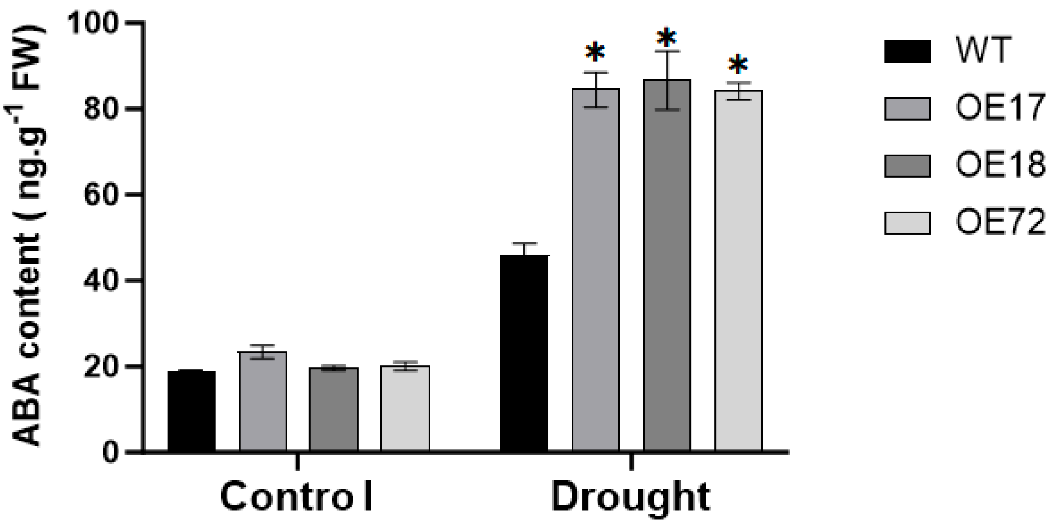


**Figure S1.** ABA levels in *SsDHN* overexpression plants. Data represent the mean ± SD (n = 3, * $p < 0.05$ by Student's *t*-test).
